# Bioinspired Polydopamine Coatings Facilitate Attachment of Antimicrobial Peptidomimetics with Broad-Spectrum Antibacterial Activity

**DOI:** 10.3390/ijms23062952

**Published:** 2022-03-09

**Authors:** Katrina Browne, Rajesh Kuppusamy, Renxun Chen, Mark D. P. Willcox, William R. Walsh, David StC. Black, Naresh Kumar

**Affiliations:** 1 School of Chemistry, University of New South Wales (UNSW) Sydney, Sydney 2052, Australia; k.browne@unsw.edu.au (K.B.); r.kuppusamy@unsw.edu.au (R.K.); r.chen@unsw.edu.au (R.C.); 2 Surgical and Orthopaedic Research Laboratories (SORL), Prince of Wales Clinical School, Prince of Wales Hospital, University of New South Wales (UNSW), Randwick 2031, Australia; w.walsh@unsw.edu.au; 3 School of Optometry and Vision Science, University of New South Wales (UNSW) Sydney, Sydney 2052, Australia; m.willcox@unsw.edu.au

**Keywords:** antimicrobial peptides, antimicrobial peptidomimetics, biofilm-mediated infections, biomaterials, hospital-acquired infections, antimicrobial coatings, polydopamine coatings

## Abstract

The prevention and treatment of biofilm-mediated infections remains an unmet clinical need for medical devices. With the increasing prevalence of antibiotic-resistant infections, it is important that novel approaches are developed to prevent biofilms forming on implantable medical devices. This study presents a versatile and simple polydopamine surface coating technique for medical devices, using a new class of antibiotics—antimicrobial peptidomimetics. Their unique mechanism of action primes them for activity against antibiotic-resistant bacteria and makes them suitable for covalent attachment to medical devices. This study assesses the anti-biofilm activity of peptidomimetics, characterises the surface chemistry of peptidomimetic coatings, quantifies the antibacterial activity of coated surfaces and assesses the biocompatibility of these coated materials. X-ray photoelectron spectroscopy and water contact angle measurements were used to confirm the chemical modification of coated surfaces. The antibacterial activity of surfaces was quantified for *S. aureus*, *E. coli* and *P. aeruginosa*, with all peptidomimetic coatings showing the complete eradication of *S. aureus* on surfaces and variable activity for Gram-negative bacteria. Scanning electron microscopy confirmed the membrane disruption mechanism of peptidomimetic coatings against *E. coli*. Furthermore, peptidomimetic surfaces did not lyse red blood cells, which suggests these surfaces may be biocompatible with biological fluids such as blood. Overall, this study provides a simple and effective antibacterial coating strategy that can be applied to biomaterials to reduce biofilm-mediated infections.

## 1. Introduction

The ability to replace and restore damaged or diseased parts of the body has remarkably improved the quality of life of many patients. However, biofilm infections on implantable medical devices bring new challenges to the field. The treatment of these infections is particularly challenging due to the unique and complex architecture of biofilms [1]. Once attached to a surface, bacteria self-produce extracellular polymeric substances and form a protective biofilm matrix [2]. These sessile bacteria undergo changes to their metabolic rate, morphology and gene expression when compared to their planktonic counterparts [3]. Within the biofilm matrix, bacteria are protected from antibiotics and the host immune response [4]. Moreover, the biofilm architecture allows for rapid horizontal gene transfer, modified gene regulation pathways and the production of antibiotic-destroying enzymes [5]. Mature biofilms are up to 5000× more resistant to antibiotics, which exceeds the clinical therapeutic capacity of these compounds [6]. Once a biofilm infection is identified, treatment options are limited, often requiring the surgical removal of the infected device and aggressive antibiotic therapy to clear infection in the surrounding tissues [7,8]. The entire treatment process is costly both in time and money. In the United States alone, more than 1.7 million infections per year are biofilm-mediated, costing approximately 94 billion USD [9,10].

Current approaches have had limited success in preventing biofilms from forming on medical devices. The most effective approach is prophylactic antibiotic therapy 1–2 h prior to surgical incision and antibiotic treatment for 24 h following surgery [11]. However, the spread of antibiotic-resistant bacteria is rising [12,13]. Additionally, bacteria can remain in a lowered metabolic state in the presence of antibiotics, only to flourish when the antibiotic treatment is stopped [14]. In orthopaedic surgery for prosthetic joint replacement, an antibiotic-loaded cement is often used [15]. However, the clinical benefit remains controversial. In 2020, a review of clinical evidence revealed critical concerns around the use of antibiotic-loaded cements [16]. There was a substantial lack of evidence demonstrating efficacy. Of particular concern was the fact that the antibiotic formulations were released in an initial burst, leaving only sub-therapeutic concentrations released in the following days [17,18,19]. This has been shown to promote the development of antibiotic-resistant bacteria [20]. Different approaches are being developed to prevent initial biofilm formation on medical devices, ranging from novel drug delivery systems, surface modification and the use of novel antimicrobial compounds. The current study combines the latter two, creating an antimicrobial surface coating using novel antimicrobial compounds.

Antimicrobial peptides are natural products and show great therapeutic potential as potent, broad-spectrum antimicrobials. The clinical potential of this new class of antibiotic was recently reviewed [21]. In summary, their unique mechanism of action provides a novel approach to overcoming antibiotic-resistant infections. Many antimicrobial peptides act on the bacterial cell membrane rather than intracellular targets, where they disturb membrane integrity and result in rapid cell lysis [21]. As these compounds do not need to be internalised by bacteria to exert their antibacterial activity, they may be immobilised onto a surface and maintain activity. This unique property adds another dimension to their clinical potential [22,23,24]. While natural antimicrobial peptides show promise in the research stage, peptide-based drugs present challenges when used clinically [25]. Peptides are more difficult to synthesise and more susceptible to proteases compared to other drug classes [26,27]. Peptidomimetics are rationally designed compounds that mimic the biological function of peptides while overcoming many of the obstacles for their production and clinical use [28]. They typically arise either from the modification of an existing peptide or from designing similar systems that mimic peptides [29]. Our research group has developed various peptidomimetics with promising antibacterial and antibiofilm activity [30,31,32,33].

The aim of this study was to covalently attach three antimicrobial peptidomimetics to biomaterial surfaces, using dopamine as a versatile linking agent. We hypothesised that antimicrobial peptidomimetics would retain activity when covalently attached to a surface and that these surfaces would have lower cytotoxic effects compared to free antimicrobial peptidomimetics. The attachment of peptidomimetics to biomaterial surfaces was facilitated by ‘one-pot’ dopamine immersion coating. In the presence of oxygen and alkaline conditions, dopamine self-polymerises and forms a thin film on the surface of materials [34]. This polymerised layer has numerous catechol groups allowing the covalent attachment of thiol or amine groups [35]. This simple and versatile coating method has been used to attach antimicrobial peptides to a variety of biomaterial surfaces, including titanium, glass, plastics, polymers, gauze and stainless steel [36,37,38]. Covalently attaching peptidomimetics using dopamine-functionalised surfaces has significant potential for these coatings to be applied clinically to medical devices. Effective antimicrobial and biocompatible coatings offer a promising strategy to prevent biofilm-mediated infections.

## 2. Results

### 2.1. Biofilm Activity of Peptidomimetics

**Figure 1:**
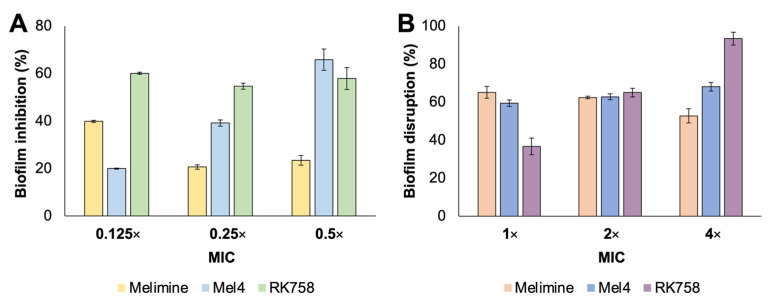
Activity of peptidomimetics against *Staphylococcus aureus* 38 biofilms. (**A**) Biofilm inhibition when peptidomimetics are used at sub-MIC. (**B**) Disruption of pre-formed biofilms when peptidomimetics are used ≥MIC. Data represent mean ± SD, *n* = 2. MIC = minimum inhibitory concentration.

The three peptidomimetics, Melimine, Mel4 and RK758, were selected as lead compounds due to their activity against clinically relevant bacterial isolates. Two *S. aureus* 38 biofilm assays were conducted to determine if they would be suitable as antibiofilm compounds, and thus prevent biofilms forming on surfaces. All peptidomimetics inhibited biofilm formation when used below their MIC (Figure 1A). RK758 showed 55–60% inhibition of biofilms between 0.5× and 0.125× MIC, whereas biofilm inhibition decreased as the concentration of Mel4 decreased, from 66% to 20% (Figure 1A). The largest molecular weight compound, Melimine, had the lowest overall biofilm inhibition ranging from 21% to 40% (Figure 1A). When peptidomimetics were administered to pre-formed biofilms, RK758 had the highest biofilm disruption peaking at 93% reduction in biomass at 4× MIC (Figure 1B). The biofilm disruption of peptidomimetics Melimine and Mel4 ranged between 65–53% and 68–60%, respectively (Figure 1B).

### 2.2. X-ray Photoelectron Spectroscopy (XPS)

Peptidomimetics were attached to glass surfaces, and the elemental composition of different surfaces was evaluated with XPS (Table 1). The seven treatment groups reflect various stages and compounds in the coating procedure. There was no statistical difference between untreated glass surfaces and glass that had been incubated with NaHCO_3_ buffer (Table 1). When glass was incubated with 0.25 mg*mL^−1^ dopamine hydrochloride in NaHCO_3_ buffer for 24 h, there was an increase in % C and N on the surface and a reduction in O, Na and Si (Table 1). For the ciprofloxacin treatment group, there were only minor changes to the surface composition, with no F detected (Table 1). For Melimine, Mel4 and RK758 treatment groups, there was an increase in % C and N on the surface and a reduction in O, Na and Si compared to polydopamine alone (Table 1).

### 2.3. Water Contact Angle

**Figure 2:**
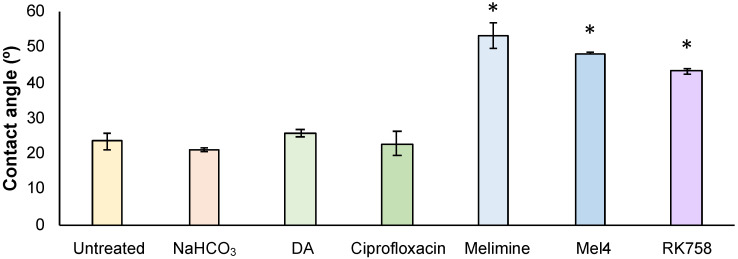
Water contact angle of treated surfaces. Data represent mean ± SD, *n* = 3. DA = polydopamine. Statistical analysis was conducted on GraphPad Prism v9 using a one-way ANOVA with Dunnett’s multiple comparisons test. * represents statistical difference to DA treatment group, *p* < 0.05.

The surface modifications were further confirmed using a goniometer to calculate water contact angle. Compared to untreated glass, there was no significant difference in the water contact angle of NaHCO_3_-buffer-, polydopamine- and ciprofloxacin-treated surfaces (Figure 2). There was a significant increase in the water contact angle for Melimine-, Mel4- and RK758-treated surfaces (Figure 2).

### 2.4. Antibacterial Activity of Surfaces

**Figure 3:**
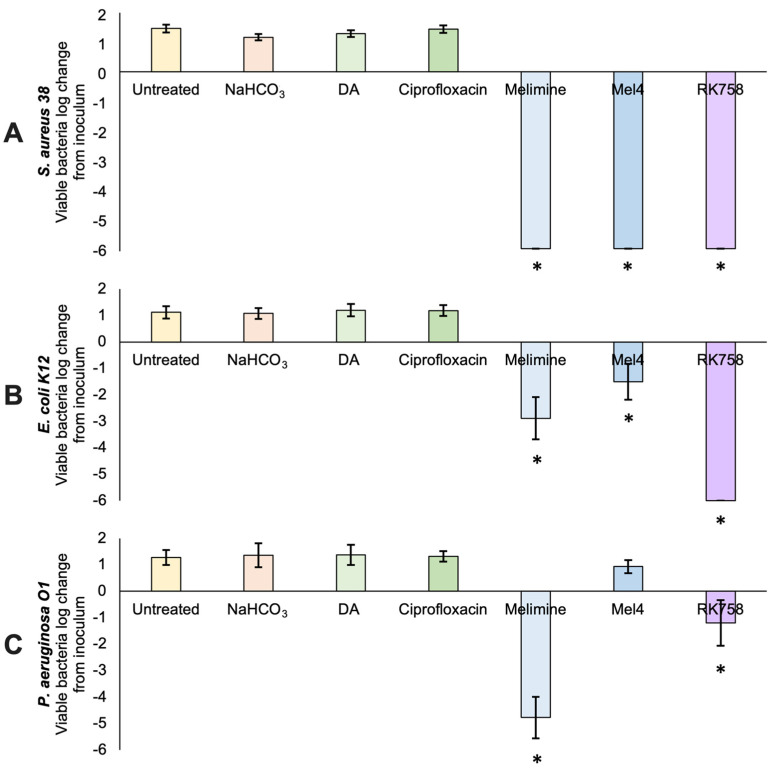
Antibacterial activity of coated surfaces. (**A**) *Staphylococcus aureus* 38. (**B**) *Escherichia coli* K12. (**C**) *Pseudomonas aeruginosa* O1. Data represent mean ± SD, *n* = 3. DA = polydopamine. Statistical analysis was conducted on GraphPad Prism v9 using a one-way ANOVA with Dunnett’s multiple comparisons test. * represents statistical difference to polydopamine (DA) treatment group, *p* < 0.05.

The antibacterial activity of polydopamine-coated surfaces against *S. aureus*, *E. coli* and *P. aeruginosa* was assessed following the 22196:2011 protocol (Figure 3). For all bacteria tested, the control groups (untreated glass, NaHCO_3_ buffer, polydopamine and ciprofloxacin) showed an approximate 1 log increase in the number of viable bacteria on the surface after 24 h incubation compared to the original inoculum (~10^5^ CFUmL^−1^). When the peptidomimetics (Melimine, Mel4 and RK758) were covalently attached to the surface using polydopamine, there were 0 viable *S. aureus* bacteria that could be recovered from the surface (Figure 3A). This equates to a 6 log reduction in the number of viable bacteria on the surface compared to the initial inoculum loaded onto the surface. The peptidomimetic coatings Melimine and Mel4 showed 2.9 and 1.5 reductions in *E. coli* on the surface after 24 h (Figure 3B). The most active coating against *E. coli* was covalently attached RK758, with the complete eradication of bacteria, or a 6 log reduction in bacteria loaded onto the surface (Figure 3B). The most active coating against *P. aeruginosa* was covalently attached Melimine, with a 4.8 log reduction in the number of viable bacteria on the surface (Figure 3C). The peptidomimetic Mel4 showed no activity against *P. aeruginosa* (Figure 3C). The peptidomimetic RK758 showed a 1.2 log reduction in the number of viable *P. aeruginosa* bacteria on the surface (Figure 3C). Compared to the dopamine surface coatings, there was a significant reduction (*p* < 0.05) in the number of bacteria recovered from the Melimine-, Mel4- and RK758-coated surfaces, except for the Mel4 group when *P. aeruginosa* was used (Figure 3A).

### 2.5. Scanning Electron Microscopy (SEM)

**Figure 4:**
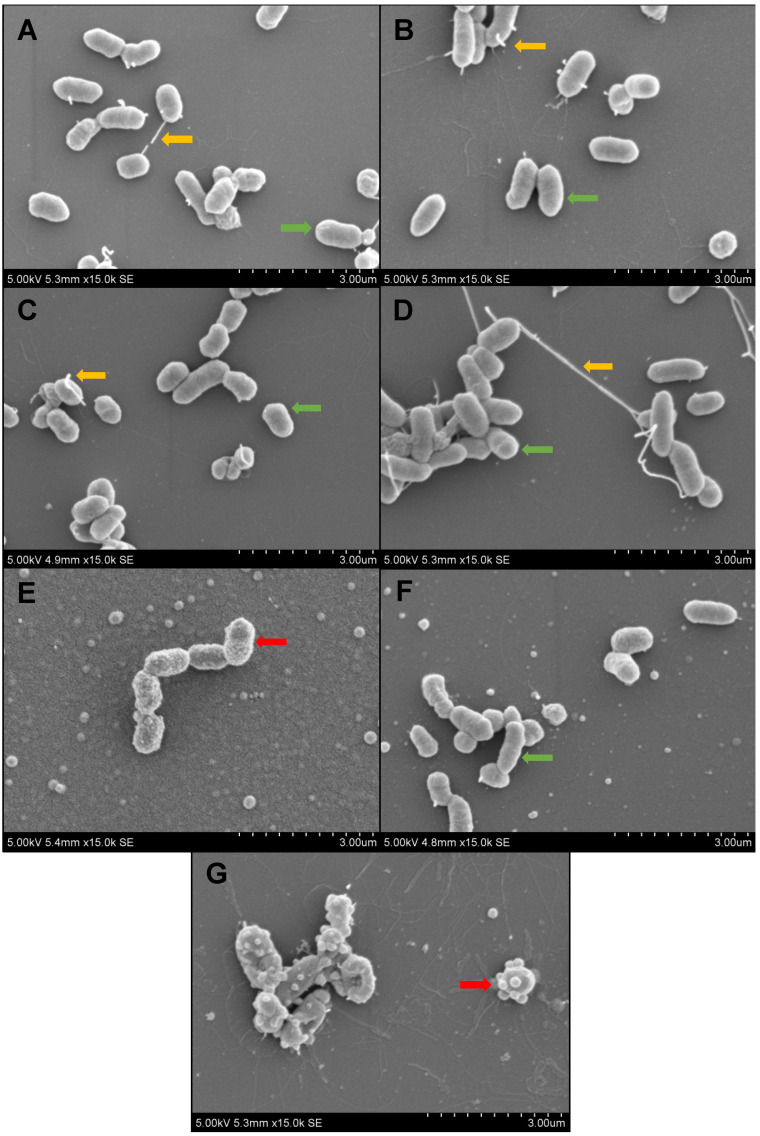
Scanning electron microscopy of *E. coli* K12 membrane morphology on (**A**) untreated glass, (**B**) NaHCO_3_ buffer, (**C**) polydopamine, (**D**) ciprofloxacin, (**E**) Melimine, (**F**) Mel4 and (**G**) RK758. Yellow arrows show normal cellular projections. Green arrows show smooth membrane morphology. Red arrows show abnormal membrane morphology.

SEM was used to further examine the mechanism of antibacterial activity of coated surfaces. *E. coli K12* was used as this species is known to show distinct morphological changes when exposed to antimicrobial peptides. For control groups (untreated glass, NaHCO_3_ buffer, polydopamine and ciprofloxacin) the outer membranes of *E. coli* showed expected morphology (Figure 4A–D). Bacteria show smooth, uniform membranes (green arrows) and many have cellular projections (yellow arrows). Melimine-coated surfaces resulted in altered membrane morphology, with the *E. coli* outer membrane having a distinct ‘ruffled’ appearance (Figure 4E). Mel4-coated surfaces showed *E. coli* membranes with subtle changes in morphology. While the membranes appeared normal at low magnification, at 15,000× magnification, a slight ruffling of the membrane morphology was visible (Figure 4F). The most distinct change in membrane morphology was seen in the RK758-coated surfaces. These membranes showed severe membrane blebbing and deflated cell membranes (Figure 4G).

### 2.6. Haemolysis Assay for Coated Surfaces

A haemolysis assay was used to determine if red blood cells would lyse upon contact with polydopamine-coated surfaces. OD_540_ data are presented as the mean of duplicate samples ± SD. Compared to the negative control, there was significant haemolysis of red blood cells when incubated in Milli-Q water (*p* = 0.0002) (Table 2). For all surface coating groups, the % haemolysis was below %1, with NaHCO_3_, Melimine and RK758 surfaces showing absorbance readings below the negative control (Table 2). Directly compared to the negative control, all surface coatings had *p* values > 0.05, indicating no significant haemolysis (Table 2).

### 2.7. Leaching of Peptidomimetics from Surfaces

After 24 h of incubation of coated samples in PBS, the supernatant was isolated and assessed via UV spectroscopy to detect for the leaching of compounds. The peak absorbance of dopamine, Melimine and Mel4 was 280 nm, and RK758 was 360 nm. At these wavelengths, the OD reading was 0 for all samples. Furthermore, when the supernatants were recovered after 24 h of incubation in PBS and after being inoculated with *S. aureus* 38, there was no inhibition of bacterial growth.

## 3. Discussion

Biofilm formation is a critical factor for medical-device infections and is exacerbated by the failing activity of commercial antibiotics. Therefore, novel-acting antibiotics and new applications are necessary to prevent biofilms forming on medical devices. Our study aimed to attach antimicrobial peptidomimetics to surfaces via polydopamine covalent attachment. We demonstrated the antibiofilm activity of these compounds, their retained activity after attachment to surfaces and their biocompatibility with blood cells.

The peptidomimetics Melimine, Mel4 and RK758 were chosen for this study due to their broad-spectrum antibacterial activity, including multidrug-resistant isolates [39,40]. When used in solution as free compounds, the peptidomimetics all displayed anti-biofilm activity (Figure 1). We found the ‘one-pot’ polydopamine coating method facilitated the attachment of these peptidomimetics to surfaces—and retained their antibacterial activity when attached to surfaces. To confirm and characterise the surface modification, we conducted XPS analysis. The elemental composition of glass surfaces did not change between untreated and buffer-only treatment groups (Table 1). Thus, the buffer alone did not alter the surface composition. When dopamine was added to the buffer solution, there was a sharp increase in the percentage of C and N on the surface (Table 1). This can be attributed to a polydopamine layer forming on the material surface [41,42]. When the antibiotic ciprofloxacin was added to the dopamine solution, there was no detection of F on the surface, suggesting that ciprofloxacin was not covalently attached to the surface, and any loosely bound or absorbed compound was removed in the washing process (Table 1). As the lowest MIC of ciprofloxacin is 0.5 µg mL^−1^ (*S. aureus* 38) and the surfaces were coated with a 4 mg*mL^−1^ concentration of ciprofloxacin, the absence of antibacterial activity data validate the washing procedure [43]. In contrast, when the peptidomimetics Melimine, Mel4 and RK758 were added to the dopamine solution, there was an increase in the percentage of C and N (Table 1). For the RK758 treatment group, Br was detected on the surface, which is only present in the RK758 compound (Table 1, Scheme S1 available in Supplementary Material). This supports the hypothesis that that the peptidomimetics are incorporated into the polydopamine matrix. The C/N ratio can be used to predict the peptide attachment to surfaces; for untreated surfaces, this ratio is 57.7, which decreases to 9.1 for polydopamine, 2.7 for Melimine, 2.9 for Mel4 and 4.6 for RK758. The increase in N on surfaces can be attributed to the dopamine and peptidomimetic chemical structures. The largest molecule, Melimine, had the highest N content—which is reflected in the C/N ratio.

The water contact angles of surfaces were measured to gain further insight into the chemical properties of coated surfaces. Contact angles between untreated glass, buffer, polydopamine and ciprofloxacin treatment groups did not significantly differ (Figure 2). However, for all peptidomimetic treatment groups, the water contact angle increased significantly (Figure 2). This demonstrates that the peptidomimetic coatings increased the hydrophobicity of surfaces. Water contact angle can be used as an indicator for biocompatibility [44]. Comparing our peptidomimetic data set to literature reports, water contact angles between 52° and 58° had the highest compatibility with 95% fibroblast adhesion after 2 h, compared to 60% adhesion for untreated glass [45]. Peptidomimetic attachment increased the hydrophobicity of surfaces, with Melimine lying within this range (Figure 2). Thus, these peptidomimetic coatings may provide the best scaffold for implantable devices that require host cell tissue integration such as prosthetic devices. Further studies are needed to assess the biocompatibility of coated surfaces with host cells.

After the chemical characterisation of the coatings, we quantified the antibacterial activity of surfaces. When surfaces were challenged with the Gram-positive bacteria, *S. aureus*, there was the complete eradication of bacteria for all peptidomimetic groups (Figure 3A). The buffer, dopamine and ciprofloxacin treatment groups all showed similar counts of bacteria recovered from the surface (Figure 3). This suggests that these surfaces had no antibacterial activity and that the peptidomimetic functionalisation is indeed responsible for bacterial eradication. The ciprofloxacin group had no antibacterial activity, confirming the XPS data that ciprofloxacin did not attach and was effectively washed from surfaces (Figure 3). As the washing procedure was effective, we suggest that the antibacterial activity seen in peptidomimetic groups is from the covalently attached peptidomimetics and not residual peptidomimetic unbound on surfaces. To confirm this hypothesis, a leaching assay was conducted to detect any peptidomimetic that had detached from the surface. No dopamine or peptidomimetic was detected in the supernatant of surfaces after 24 h. Additionally, the supernatant showed no antibacterial activity when inoculated with *S. aureus*. This is consistent with other polydopamine coatings that showed minimal leaching after prolonged incubation [46].

However, for surfaces challenged with Gram-negative *E. coli*, only RK758 showed the complete eradication of bacteria (Figure 3B). Conversely, RK758 showed low activity against *P. aeruginosa* (Figure 3C). Paradoxically, the Melimine coating showed moderate activity against *E. coli* and high activity against *P. aeruginosa* (Figure 3B,C). The shortened Melimine derivative, Mel4, did not show the expected activity when attached to surfaces. Mel4 had low activity against *E. coli* and no activity against *P. aeruginosa* (Figure 3B,C). This differs from previous work where Mel4 was shown to be highly effective in killing *P. aeruginosa* when coupled to glass surfaces via EDC coupling [47]. In the study by Yasir et. al., Melimine and Mel4 were attached to glass surfaces with the random attachment at any of the amine groups to 4-azidobenzoic acid [47]. The antibacterial activity of Melimine- and Mel4-coated surfaces was monitored from 15 min to 90 min and showed a rapid decrease in the viability of surface-attached *P. aeruginosa*. After 90 min, Melimine- and Mel4-coated surfaces killed 82% and 63% of surface-attached bacteria, respectively [47]. The current study differs in methodology and assesses antibacterial activity after 24 h incubation. One hypothesis is that after 90 min, the remaining viable bacteria can repopulate the surface. Alternatively, the concentration of Melimine and Mel4 attached via dopamine attachment is not comparable with the concentration attached via EDC coupling. Further studies into the time-to-kill kinetics and the concentration of peptidomimetics attached to surfaces will elucidate these unknowns.

To our knowledge, this is the first study to quantify *E. coli* viability on Melimine, Mel4 and RK758 surfaces. When each peptidomimetic was used at the same coating concentration (4 mg*mL^−1^), each demonstrated varying activity against different bacterial strains. This is likely due to the amount of peptidomimetic attached, complex interactions between different moieties of the peptidomimetics and the structural confirmation of active moieties when incorporated into the polydopamine matrix [48]. It is unlikely that Melimine and Mel4 attach to the dopamine matrix via the N-terminal poly-arginine region, given that this region is essential for broad-spectrum activity [49]. The three lysine residues are also required for activity against *S. aureus*, and thus, we predict Melimine and Mel4 are not attached via these residues [49]. RK758 showed the greatest activity against *S. aureus* and *E. coli* but low activity against *P. aeruginosa*. We hypothesise that the coatings have dose-dependent activity, similar to their MIC in solution, which is correlated to the concentration of attached peptidomimetic.

In a previous study by Chen et al., Melimine was tethered to malemide-functionalised titanium surfaces. These surfaces reduced biofilm formation by 84% for *S. auerus* and 62% for *P. aeruginosa* [48]. Furthermore, animal models showed a 2 log reduction in the number of bacteria in vivo. In the present study, dopamine facilitated peptidomimetic attachment to surfaces where there was a 100% reduction in biofilm formation for *S. aureus* (Figure 3). While both attachment methods demonstrate antibacterial activity, polydopamine surface coatings may be useful for biomaterial surfaces that are unsuitable for malemide functionalisation.

In this study, the XPS detection of Si on surfaces provides insight into the surface coverage of different treatment groups (Table 1). Most notably, the peptidomimetic groups have less than 1% Si on surfaces, suggesting that dopamine–peptidomimetic coatings cover the majority of the material surface (Table 1). In a recent study by Hasan et al., 2020, an antimicrobial peptidomimetic was immobilised on surfaces via a 2kDA PEG tether [50]. The biofouling activity of surfaces was assessed using *P. aeruginosa*. Their findings established that the density of peptidomimetics on surfaces was not as important as spatial separation between attached peptidomimetics. The number of live bacteria on peptidomimetic-attached surfaces was similar to the Melimine surface coating in this study for *P. aeruginosa*. Further studies to determine the spatial organisation of these peptidomimetics would be beneficial in determining the intricacies of their mechanism of action.

To elucidate the mechanism of action of surface-attached peptidomimetics, we used SEM to visualise the morphology of *E. coli* on surfaces. This bacteria was chosen as *E. coli* cell membranes are known to show subtle changes in morphology [51]. Untreated, buffer-, dopamine- and ciprofloxacin-treated surfaces showed the expected *E. coli* morphology (Figure 4). Normal cellular projections and smooth bacterial membranes were seen for *E. coli* on control surfaces (Figure 4A–D). Melimine-coated surfaces showed ruffled membrane morphology, indicative of membrane damage (Figure 4E). Mel4-coated surfaces showed minimal membrane damage but a lack of cellular projections (Figure 4F). Conversely, RK758-coated surfaces showed severe membrane blebbing, indicative of intracellular components leaking or exocytosed from the cell (Figure 4G). Overall, the SEM images complement the surface-activity data and suggest that covalently attached peptidomimetics exert their antibacterial activity via interaction with and disruption of the bacterial cell membrane. Moreover, the mechanism of peptidomimetic activity is dependent on the chemical structure and orientation in the polydopamine matrix, as seen by varying membrane morphologies of bacteria on the peptidomimetic coatings.

A haemolysis assay was used to quantify the lysis of red blood cells upon contact with polydopamine coated surfaces. A haemolysis assay is considered a preliminary measure of toxicity. Many free peptides are haemolytic at high concentrations due to their ionic interaction with red blood cells [52,53]. Compared to the negative control, there was no haemolysis of red blood cells for any of the coated surfaces (Table 2). This is an important consideration for biomaterials that are in contact with bodily fluids, specifically blood. Horse RBCs were used in this study and should be considered as a limitation when interpreting results. While animal models are used extensively in research to determine potential mammalian cell toxicity, there are molecular intricacies of human red blood cells that cannot be adequately replicated.

In a separate clinical trial, the antimicrobial peptide Melimine was covalently attached to contact lenses (ACTRN 12613000369729) via EDC (1-ethyl-3-[3-dimethylaminopropyl] carbodiimide hydrochloride) coupling, rather than polydopamine. It was noted that Melimine-coated lenses produced corneal staining in some patients [54]. When the Mel4 peptide was attached to contact lenses, there was no change in the ocular surface physiology (including corneal staining) during extended contact lens wear for 14 days (ACTRN1261500072556) [39]. These studies demonstrate the need to fully evaluate surface coatings for clinical use. Further toxicity studies are needed to evaluate the clinical potential of these polydopamine coatings, but preliminary data are promising for their biocompatibility.

This study aimed to generate simple and effective antibacterial coatings. Many studies have assessed the antibacterial activity of free peptides in solution; however, few have investigated the activity and mechanism of action of surface-attached peptidomimetics. This study has shown that these attached peptidomimetics are able to kill bacteria on contact, yet further research is required to fully comprehend the complex structure activity relationships of surface-attached peptidomimetics and bacterial cell membranes. A limitation of this study is the inability to quantify the precise amount of peptidomimetic attached to the surface. Determination of the concentration of attached peptidomimetics is necessary to proceed in clinical development. Furthermore, a comparison of the different behaviour of each peptidomimetic against a variety of bacterial strains will further elucidate the mechanism of action and provide insight into the potential clinical use of these coatings. Animal models to demonstrate biocompatibility would propel these coatings into clinical trials as potential biofilm-mitigating coatings for medical devices.

In summary, this study demonstrated that dopamine is a simple, versatile linking agent to attach peptidomimetics to surfaces. Melimine-, Mel4- and RK758-tethered peptidomimetics showed the complete eradication of Gram-positive *S. aureus* and had varying activity against Gram-negative bacteria *E. coli* and *P. aeruginosa*. The optimisation of these surface coatings could have profound therapeutic implications, as they address the unmet clinical need of biocompatible and anti-biofilm coatings.

## 4. Materials and Methods

### 4.1. Reagents

Phosphate-buffered saline (PBS) was prepared (NaCl 8 g/L, KCl 0.2 g/L, Na_2_HPO_4_ 1.4 g/L and KH_2_PO_4_ 0.24 g/L; pH 7.4) and autoclaved prior to use. Milli-Q water (18.2 MΩ cm) from Millipore Co. (Burlington, MA, USA) was autoclaved prior to use. Dopamine hydrochloride, ciprofloxacin, 1% crystal violet solution (*w*/*v*), 70% (*w*/*v*) ethanol, Dey Engley (D/E) neutralising broth and sodium bicarbonate (NaHCO_3_) were purchased from Sigma Aldrich (Burlington, MA, USA). NaHCO_3_ buffer was prepared in Milli-Q water, pH adjusted to 8.5 and autoclaved prior to use. Mueller–Hinton Broth (MHB) and tryptic soy agar (TSA) were purchased from Oxoid (Basingstoke, UK), prepared in Milli-Q water, pH adjusted to 7 and autoclaved prior to use. Defibrinated horse red blood cells (RBCs) were purchased from Edwards Group Pty Ltd. (Narellan, Australia).

### 4.2. Bacterial Strains and Culture

Three common clinical pathogens were used in this study: Staphylococcus aureus 38, Escherichia coli K12 and Pseudomonas aeruginosa O1. *S. aureus* 38 and *P. aeruginosa* O1 were isolated from clinical samples, and *E. coli* K12 is a frequently used laboratory strain. All strains were cultured as follows: a single bacterial colony was cultured in MHB for 18 h in a humidified incubator at 37 °C. After centrifugation, cells were resuspended in MHB to ~10^5^ CFU mL^−1^ for antibacterial activity assays and ~10^6^ CFU mL^−1^ for biofilm assays and scanning electron microscopy (SEM).

### 4.3. Peptidomimetic Design and Synthesis

The peptidomimetic Melimine (TLISWIKNKRKQRPRVSRRRRRRGGRRRR) is a chimera of the naturally occurring peptides melittin (GIGAVLKVLTTGLPALISWIKRKRQQ) and protamine (MPRRRRSSSRPVRRR-RRPRVSRRRRRRGGRRRR) [55]. A derivative of Melimine, Mel4 (KNKRKRRRRRRGGRRRR) was developed for improved biocompatibility [39]. These peptides were purchased from AusPep Peptide Company (Tullamarine, VIC, Australia). The purity of the purchased peptides was ≥90%. The peptidomimetic compound RK758 used in this study was synthesised according to the patents (WO2018081869A1 and Australian Provisional Patent Application No. 2021902457.), and its chemical structure is available in the Supplementary File.

### 4.4. Inhibition of Biofilm Formation

The ability of peptidomimetics to inhibit biofilm formation was measured using a crystal violet staining assay to quantify biomass. *S. aureus* 38 was chosen due to the strong biofilm-forming ability of this strain. Bacteria were grown for 18 h, as described in Section 4.2, before being diluted in MHB to a final inoculum of ~10^6^ CFU mL^−1^. A total of 50 µL of bacterial solution was added to each well of a 96-well plate (COSTAR, Corning Incorporated, New York, NY, USA) and mixed in equal volume of peptidomimetic solution. The final well concentrations of peptidomimetics were 1×, 0.5×, 0.25× and 0.125× MIC. The 1× MIC treatment group was used as a positive control to confirm the absence of biofilm formation. Following 18 h of incubation at 37 °C, cultured media were carefully aspirated and washed with 125 µL of Milli-Q water to remove any loosely bound bacteria and residual peptidomimetic. Adherent biofilms were stained with 125 µL of 0.1% (*w*/*v*) crystal violet solution at room temperature. After 10 min, wells were washed three times with 200 µL of Milli-Q water. An amount of 200 µL of 70% (*w*/*v*) ethanol was added to each well to solubilise the crystal violet. Optical density was measured using a spectrophotometer to quantify the absorbance of each well at 595 nm. Biofilm inhibition was calculated as a percentage compared to positive control wells using the following formula [56]. Data are expressed as the mean ± SD of two independent experiments, performed in triplicate technical replicates.

$$\mathrm{Change}\;\mathrm{in}\;\mathrm{biomass}\;\left(\%\right)=\frac{{\mathrm{OD}}_{\mathrm{positive}\;\mathrm{control}}-{\;\mathrm{OD}}_{\mathrm{sample}}}{{\mathrm{OD}}_{\mathrm{positive}\;\mathrm{control}}}\;\times \;100$$

### 4.5. Disruption of Pre-Formed Biofilms

The disruption of pre-formed biofilms upon exposure to peptidomimetics was assessed using the crystal violet staining assay. *S. aureus* 38 was chosen due to the strong biofilm-forming ability of this strain. Bacteria were grown for 18 h, as described in Section 4.2, before being diluted in MHB to a final inoculum of ~10^6^ CFU mL^−1^. A total of 100 µL of bacterial solution was added to each well of a 96-well plate (COSTAR, Corning Incorporated, New York, NY, USA) and incubated for 18 h at 37 °C to establish biofilms. Media were then carefully aspirated, and wells were washed with 125 µL of Milli-Q water to remove any loosely bound bacteria. Peptidomimetics were prepared in MHB to final well concentrations of 1×, 2× and 4× MIC. An amount of 100 µL of each peptidomimetic solution was added to each well, and plates were incubated at 37 °C for 18 h. Wells were then prepared and stained, and biomass quantification was assessed as described above. Data are expressed as the mean ± SD of two independent experiments, performed in triplicate technical replicates.

### 4.6. Polydopamine Attachment to Surfaces

**Figure 5:**
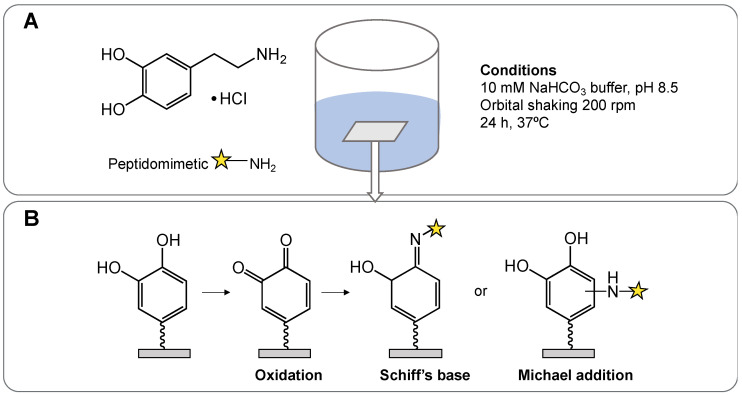
Polydopamine attachment of peptidomimetics to surfaces. (**A**) One-pot polydopamine–peptidomimetic surface coating. (**B**) Predicted reactions between surface-attached polydopamine functionalities and peptidomimetic amine moieties.

A ‘one-pot’ polydopamine coating method was used as a linking substrate to attach antimicrobial peptidomimetics to glass (Figure 5). In this method, 0.25 mg*mL^−1^ dopamine hydrochloride and 4 mg*mL^−1^ peptidomimetic were simultaneously dissolved in 10 mM NaHCO_3_ buffer, pH 8.5 [41]. Treatment groups and coating reagents are listed in Table 3. Glass coverslips (25 mm diameter) were then immersed in 2 mL of this solution (Table 3) and incubated at 37 °C with orbital shaking (200 rpm) for 24 h. Materials were then washed thoroughly with Milli-Q water to remove any loosely bound compound from the surface. The fluroquinolone antibiotic, ciprofloxacin (4 mg*mL^−1^), was used as a negative control, as this compound must be internalised by the bacterium, and the antibacterial activity in this treatment group would reflect compound leaching from the surface coating [57]. The untreated glass surface controls were hydrated in PBS under the same conditions. The proposed mechanism of attachment is provided in Figure 5, where an amine group of the peptidomimetic reacts with the dopamine-functionalised surface.

### 4.7. X-ray Photoelectron Spectroscopy (XPS)

XPS was used to quantify the elemental composition of surfaces (ESCALAB250Xi, Thermo Scientific, Waltham, MA, USA). The X-ray source was monochromated Al Kα, and the photo-energy was 1486.68 eV with a source power of 160 W (14.5 × 11 mA). The background vacuum was better than 2E-9 mbar.

### 4.8. Water Contact Angle

Surface wettability was evaluated using a contact angle goniometer (Model no. 500 Rame-Hart, Inc. Randolph, NJ, USA). Using the sessile drop method, a 3 µL drop of Milli-Q water was placed onto the surface. The static contact angle was measured using the DROP image advanced software (v3, Rame-Hart, Inc. Randolph, NJ, USA) to calculate the water contact angle. Data are presented as the mean ± SD of three independent experiments. Statistical analysis was conducted on GraphPad Prism (v9, GraphPad, San Diego, CA, USA) using a one-way ANOVA with Dunnett’s multiple comparisons test. The significance was set below *p* = 0.05.

### 4.9. Antibacterial Activity of Surfaces

The protocol for the measurement of antibacterial activity on antimicrobial surfaces was obtained from the International Standards Organisation (Geneva, Switzerland), ISO 221961:2011 and performed with minor adjustments. Briefly, surfaces were prepared in triplicate and UV-C sterilised prior to biological testing. In independent experiments, *S. aureus*, *E. coli* and *P. aeruginosa* were prepared in 1:500 MHB:PBS and diluted to a final density of 180 cells per mm^2^ surface area of the sample. Samples were then statically incubated at 37 °C in a humidified chamber for 24 h. The same volume of bacterial inoculum was pour-plated with TSA and used to calculate the number of bacterial cells added to the surfaces. Surfaces were vortexed to remove adherent bacteria from the surface. Recovered sample fluids underwent 1:10 serial dilutions in D/E neutralising broth and were pour-plated using TSA. Plates were incubated for 18 h at 37 °C, then the number of colonies on each plate was enumerated. Plates with 30–300 colonies were recorded, and the CFU mL^−1^ calculations were determined using the following formula. The original inoculum was used to determine log change in the number of viable bacteria on surfaces. Data are presented as the mean ± SD of three independent experiments. Statistical analysis was conducted on GraphPad Prism v9 using a one-way ANOVA with Dunnett’s multiple comparisons test. Significance was set below *p* = 0.05.

$$\mathrm{CFU}\;{\mathrm{mL}}^{-1}=\frac{{\mathrm{CFU}}_{\mathrm{plate}}\;\times \;\mathrm{dilution}\;\mathrm{factor}}{\mathrm{volume}\;\mathrm{plated}\;(\mathrm{mL})}$$

### 4.10. Scanning Electron Microscopy (SEM)

Bacterial inoculums were prepared as described for the antibacterial activity of surfaces, with an increased inoculum loaded onto surfaces (~10^6^ CFU*mL^−1^) to improve image quality. However, after 24 h incubation of bacteria, the surfaces were gently washed 3× with PBS to remove any non-adherent bacteria. Samples were then fixed in formaldehyde solution for 1 h at room temperature. Samples were then washed three times with 0.1 M sodium cacodylate buffer. Tissue processing apparatus (Pelco BioWave^®^, TedPella, Inc., Redding, CA, USA) was then used to process surfaces before critical point drying. Samples were sputter coated with Pt before being imaged with the Hitachi S3400 microscope (Hitachi, Ibaraki, Japan). SEM images were generated using 5 kV and 15,000 magnification.

### 4.11. Haemolysis Assay for Coated Surfaces

A haemolysis assay was used to quantify the lysis of defibrinated horse RBCs when exposed to coated materials. PBS was used as the non-haemolytic control, and Milli-Q water was used as the positive control for haemolysis. For this assay, 1.5 mL Eppendorf tubes were coated using the method described in ‘polydopamine attachment to surfaces’ and prepared in duplicate. Coated tubes were then washed and UV-C sterilised prior to use. A 10% RBC solution was prepared as described [58]. A total of 200 µL of RBC solution was added to each tube and incubated at 37 °C, with orbital shaking at 50 rpm, for 4 h. An amount of 100 µL of supernatant was taken from each tube and added to a 96-well plate for spectrophotometric analysis. Absorbance was measured at 540 nm and reported as OD_540_. The Milli-Q treatment group was used as the positive control for haemolysis (100%), and percentages for other samples were calculated from this value. Data are presented as % haemolysis, calculated using the following equation.

$$\%\;\mathrm{haemolysis}=\frac{{\mathrm{OD}}_{\mathrm{treatment}}{\;-\;\mathrm{OD}}_{\mathrm{negative}\;\mathrm{control}}\;}{{\mathrm{OD}}_{\mathrm{positive}\;\mathrm{control}}}\times \;100$$

As an appropriate haemolytic surface could not be tested, the OD_540_ of treatment groups was directly compared to the negative control for statistical analysis. A two-sample, independent *t*-test was used to determine whether there was a significant difference between the OD of negative control and treatment groups. A *p* value of <0.05 was considered significant, thus indicating increased haemolytic activity compared to the negative control.

### 4.12. Leaching of Peptidomimetics from Surfaces

To assess whether peptidomimetics remained attached or leached from material surfaces, a leaching assay was conducted. Surfaces were immersed in PBS for 24 h and incubated at 37 °C. UV spectroscopy was used to quantify the absorbance of dopamine, Melimine, Mel4 and RK758 in the supernatant. An aliquot of each solution was used in an MIC assay to evaluate antibacterial activity [59].

## 5. Patents

The compound RK758 used in this study is described in Australian Provisional Patent Application No. 2021902457.

## Figures and Tables

**Table 1:** Elemental composition (%) detected by X-ray photoelectron spectroscopy on glass surfaces.

Treatment Group	Elemental Composition (%)						
	C	N	O	Na	Si	Br	Other
Untreated	17.3	0.3	54.3	3.5	14.7	0	9.9
NaHCO_3_ buffer	17.0	1.5	56.3	2.2	15.1	0	7.9
Polydopamine	43.0	4.7	38.2	1.3	8.6	0	4.2
+Ciprofloxacin	37.8	3.8	40.9	2.5	10.6	0	4.4
+Melimine	60.7	22.2	17.1	0	0	0	0
+Mel4	58.2	20.2	20.3	0	0.9	0	0.4
+RK758	70.8	15.4	12.3	0.2	0.5	0.9	0

**Table 2:** Haemolytic activity of polydopamine–peptidomimetic-coated surfaces.

	NC	PC	DA	Buffer	Melimine	Mel4	RK758
OD_540_	0.124	1.785	0.137	0.108	0.118	0.130	0.105
SD	0.011	0.027	0.045	0.001	0.010	0.009	0.003
% haemolysis			0.728	−0.924	−0.336	0.308	−1.064
*p* =		0.0002	0.732	0.454	0.273	0.352	0.069

Note: NC = negative control, PC = positive control, DA = dopamine, OD = optical density, SD = standard deviation.

**Table 3:** ‘One-pot’ polydopamine coating treatment groups and reagents.

Treatment	Untreated	Buffer	DA	Ciprofloxacin	Melimine	Mel4	RK758
10 mM NaHCO_3_		X	X	X	X	X	X
DA 0.25 mg*mL^−1^			X	X	X	X	X
Ciprofloxacin 4 mg*mL^−1^				X			
Melimine 4 mg*mL^−1^					X		
Mel4 4 mg*mL^−1^						X	
RK758 4 mg*mL^−1^							X

Note: DA = dopamine hydrochloride. X denotes the conditions for each treatment group.

## Data Availability

Supplementary Material is available online.

## Supplementary Materials

The following are available online at https://www.mdpi.com/article/10.3390/ijms23062952/s1.
