# Supplementary material for: Bioinspired Polydopamine Coatings Facilitate Attachment of Antimicrobial Peptidomimetics with Broad-Spectrum Antibacterial Activity
Source: Int J Mol Sci. 2022 Mar 9;23(6):2952. doi: 10.3390/ijms23062952 (PMC8948759; doi:10.3390/ijms23062952)

Supplementary Information

# Bioinspired Polydopamine Coatings Facilitate Attachment of Antimicrobial Peptidomimetics with Broad-Spectrum Anti-bacterial Activity

**Katrina Browne [1,2], Rajesh Kuppusamy [1,3], Renxun Chen [1], Mark D. P. Willcox [3], William R. Walsh [3], David StC. Black [1,*] and Naresh Kumar [1,*]**

1 School of Chemistry, University of New South Wales (UNSW) Sydney, Sydney 2052, Australia; k.browne@unsw.edu.au (K.B.); r.kuppusamy@unsw.edu.au (R.K.); r.chen@unsw.edu.au (R.C.)

2 Surgical and Orthopaedic Research Laboratories (SORL), Prince of Wales Clinical School, Prince of Wales Hospital, University of New South Wales (UNSW), Randwick 2031, Australia; w.walsh@unsw.edu.au

3 School of Optometry and Vision Science, University of New South Wales (UNSW) Sydney, Sydney 2052, Australia; m.willcox@unsw.edu.au

* Correspondence: d.black@unsw.edu.au (D.S.B.); n.kumar@unsw.edu.au (N.K.); Tel.: +61-2-9385-4657 (D.S.B.); +61-2-9385-4698 (N.K.)

Contents:

1. General scheme for the synthesis of RK758
2. Chemical structure of ciprofloxacin

1. The general scheme for synthesis of RK-758 and the procedures are outlined in the WO2018081869A1 and Australian Provisional Patent Application No. 2021902457.
Note: RK758 contains Br and is important to consider when interpreting XPS analysis of surfaces

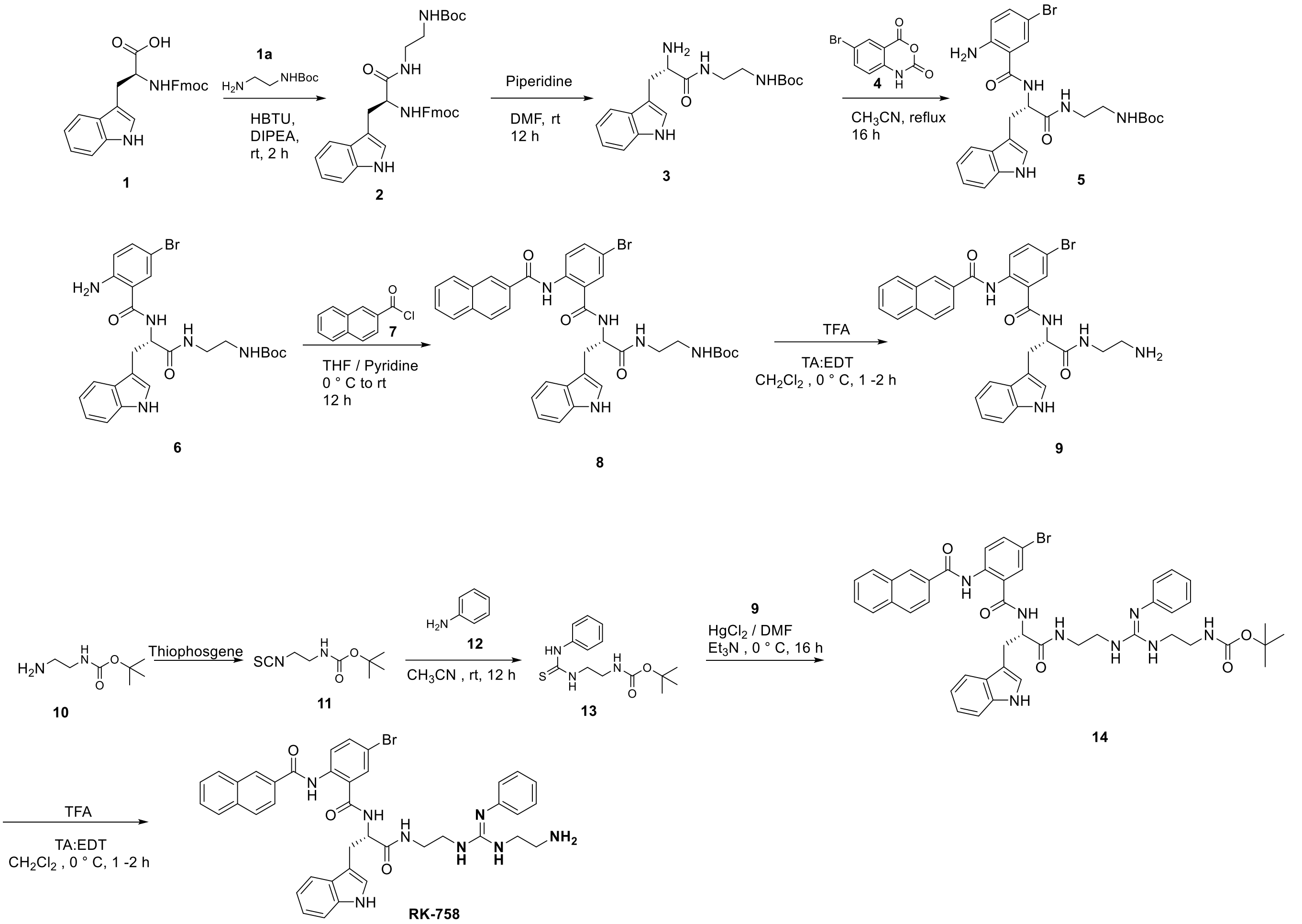


*Scheme S1: Synthetic route for RK-758*

2. Chemical structure of ciprofloxacin
Note: this compound contains F and is important to consider when interpreting XPS analysis of surfaces
Sigma Aldrich – CAS: 85721-33-1, Molecular weight: 331.34 g/mol

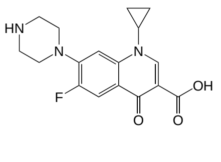

Supplement: Supplementary file 1 [file ijms-23-02952-s001.zip › ijms-1603871-supplementary.pdf]
